# Early Dietary Exposures Epigenetically Program Mammary Cancer Susceptibility through Igf1-Mediated Expansion of the Mammary Stem Cell Compartment

**DOI:** 10.3390/cells11162558

**Published:** 2022-08-17

**Authors:** Yuanning Zheng, Linjie Luo, Isabel U. Lambertz, Claudio J. Conti, Robin Fuchs-Young

**Affiliations:** 1 Department of Molecular and Cellular Medicine, Texas A&M Health Science Center, Bryan, TX 77807, USA; 2 Department of Bioengineering, Tissue Engineering and Regenerative Medicine Group (TERMeG), Universidad Carlos III de Madrid, 28903 Madrid, Spain

**Keywords:** diet, epigenetics, Igf1, mammary cancer, stem cells

## Abstract

Diet is a critical environmental factor affecting breast cancer risk, and recent evidence shows that dietary exposures during early development can affect lifetime mammary cancer susceptibility. To elucidate the underlying mechanisms, we used our established crossover feeding mouse model, where exposure to a high-fat and high-sugar (HFHS) diet during defined developmental windows determines mammary tumor incidence and latency in carcinogen-treated mice. Mammary tumor incidence is significantly increased in mice receiving a HFHS post-weaning diet (high-tumor mice, **HT**) compared to those receiving a HFHS diet during gestation (low-tumor mice, **LT**). The current study revealed that the mammary stem cell (MaSC) population was significantly increased in mammary glands from HT compared to LT mice. *Igf1* expression was increased in mammary stromal cells from HT mice, where it promoted MaSC self-renewal. The increased *Igf1* expression was induced by DNA hypomethylation of the *Igf1* Pr1 promoter, mediated by a decrease in Dnmt3b levels. Mammary tissues from HT mice also had reduced levels of Igfbp5, leading to increased bioavailability of tissue Igf1. This study provides novel insights into how early dietary exposures program mammary cancer risk, demonstrating that effective dietary intervention can reduce mammary cancer incidence.

## 1. Introduction

In 1990, Dr. David Barker proposed the fetal origins hypothesis that describes the effect of fetal nutritional status on programming subsequent disease susceptibility in the adult [1]. Since then, an increasing amount of evidence from epidemiological and experimental studies has revealed a significant association between the intrauterine environment and the development of adult diseases, including cardiovascular disease, diabetes, and cancers [2,3]. In humans, results from Dutch famine studies show that breast cancer incidence is increased by five times in women who were exposed to severe caloric restriction in utero during the 1944–1945 “hunger winter” of World War II [4].

Beyond these unfortunate circumstances in human history, in vivo dietary manipulations in rodent models allow a detailed analysis of windows of susceptibility [5], during which mammary cancer risk can be affected by diet and other environmental cues. Andrade et al. reported that the mammary cancer incidence is decreased in offspring of female rats consuming a high-fat diet during pregnancy [6]. In contrast, Aupperlee et al. demonstrated that mice exposed to a high-fat diet postweaning, starting from three weeks old and continuing throughout adulthood, have an increased mammary cancer incidence and reduced tumor latency [7]. In our previous mouse study, we showed that both the gestational and the postweaning diets are critical determinants of mammary cancer susceptibility [8]. However, the precise molecular mechanisms through which dietary exposures during defined development windows affect lifetime mammary cancer risk remain incompletely resolved.

Epigenetic modifications refer to the heritable changes in gene expression patterns and phenotypic traits that are independent of alterations in primary DNA sequence. Emerging evidence has revealed a critical role of epigenetic modifications, such as DNA methylation, in regulating cell proliferation, differentiation, and metabolism [9,10,11]. Disruption of the epigenetic programs may affect these cellular processes and contribute to breast cancer initiation and progression [12]. Hilakivi-Clarke’s group reported that prenatal exposure to a high-fat diet during gestation increases the levels of DNA methyltransferase 1 (DNMT1) in rat mammary tissues [13]. Another study by Ho’s group demonstrated that in utero exposure to a high-fat diet decreases DNMT3B levels in mammary tissues from 3-week-old rats [14].

The mammary epithelium contains multipotent mammary stem cells (MaSCs) and lineage-committed progenitors [15]. The replicative potential and cellular lifespan of stem and progenitor cells render them susceptible to the accumulation of genetic mutations [16]. Recent evidence also suggests that the transformation of mammary stem and progenitor cells leads to the formation of specific tumor subtypes [17,18,19], with different prognoses and clinical outcomes in humans [20,21]. Therefore, we hypothesized that dietary manipulations during susceptible developmental windows induce epigenetic modifications in the mammary gland, which alter the number of MaSCs, reprogram mammary cancer risk, and contribute to the determination of tumor subtypes.

## 2. Materials and Methods

### 2.1. Animal Model

**Figure 1:**
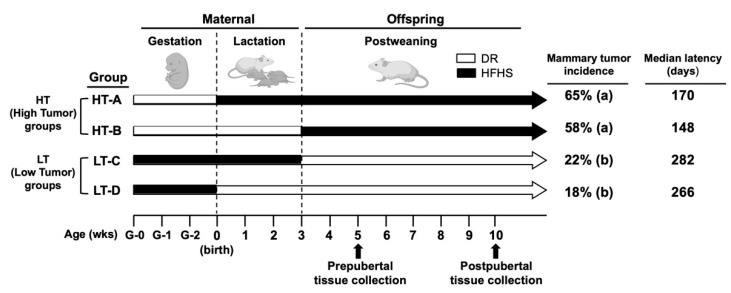
Animal model. Mice were exposed to either a DR or a HFHS diet during three developmental stages: gestation, lactation, and postweaning. Gestational and lactational diets refer to the diets delivered to the mothers. After 3 weeks of age, the pups were weaned and were maintained on the postweaning diet until death. HT-A: DR/HFHS/HFHS; HT-B: DR/DR/HFHS; LT-C: HFHS/HFHS/DR and LT-D: HFHS/DR/DR. DR: non-obesogenic, low-fat, low-sugar, and mildly restricted diet; HFHS: high-fat and high-sugar diet. Pearson’s chi-squared test was used to compare mammary tumor incidence: a ≠ b, *p* < 0.03, Ref. [8].

To investigate our hypothesis, we used our established cross-over feeding mouse model, in which SENCAR mice [22] receive either a non-obesogenic, low fat, low sugar, and mildly restricted diet (DR) or a high-fat and high-sugar (HFHS) diet during three developmental stages: gestation, lactation, and postweaning (Figure 1) [8]. In the previous study [8], we demonstrated that mammary tumor susceptibility is determined by both gestational and postweaning diets. Animals born to a mother fed a DR diet during pregnancy, nursed by a mother fed either a DR or HFHS diet, and weaned onto a HFHS diet (groups HT-A: DR/HFHS/HFHS and HT-B: DR/DR/HFHS) show the highest (average 61.5%) mammary tumor incidence and shortest tumor latency, hence referred to as high-tumor (HT) groups. In contrast, animals exposed to the HFHS diet during gestation, followed by either a DR or HFHS diet during lactation, and a DR diet postweaning (groups LT-C: HFHS/HFHS/DR and LT-D: HFHS/DR/DR) show the lowest (average 20.0%) mammary tumor incidence and longest tumor latency, hence referred to as low-tumor (LT) groups.

### 2.2. Mice and Diets

Animal maintenance and dietary treatment were performed, as described previously [8]. Briefly, SENCAR breeder mice [22] were maintained at our animal facility under a 12 h light/dark cycle at 24 °C. Defined diets were purchased from Research Diets, Inc. The cat#D01060501 was the low fat, low sugar control diet (10% calories from fat), and cat#D04011601, the high fat, high sugar (HFHS) diet (45% calories from fat). The main source of fat in the HFHS diet is lard, and diet formulations are described in Supplemental Table S1. Female breeder mice were randomized and separated into control and HFHS diet groups at four weeks of age. As these mice were sedentary, animals fed the control diet *ad libitum* typically became obese by ≥20 weeks of age [8]. To maintain a normal body weight and provide a nonobese study control, we tracked daily diet consumption of mice fed the control diet *ad libitum* and imposed a mild 12% volume reduction to the diet delivered to the age-matched mice of the diet-restricted (DR) group. DR mice received a daily allotment equal to 88% of the mean daily consumption of the *ad libtum* group, which would be similar to portion control in sedentary humans to prevent excessive weight gain [8]. This mild portion control did not result in an insufficiency of macro- or micronutrients or an increase in competition for food [8]. During lactation, DR mothers were fed the control diet *ad libitum* to ensure proper nutrition of mother and pups. HFHS animals were also provided with 10% fructose in their drinking water, simulating the effect of frequent consumption of high-fructose corn syrup sweetened beverages in humans.

HFHS mice became glucose intolerant after ten weeks compared to DR controls, as determined by glucose tolerance tests [8]. At 15 weeks of age, DR and hyperglycemic HFHS mothers were mated with 10-week-old SENCAR males. The males were maintained on normal lab chow diet and were randomized for breeding with the DR or hyperglycemic HFHS females. At birth, pups were fostered within 24 h into the lactation exposure groups. At weaning, pups were randomized into the different postweaning diet exposure groups and were maintained on that diet until sacrifice.

### 2.3. Mammary Epithelial Cell Isolation

Details for mammary epithelial cell (MEC) isolation are described in the Supplemental Materials and Methods (*Mammary Epithelial Cell Isolation*). Briefly, fourth and fifth mammary gland pairs were resected from 5-week or 10-week-old mice and were enzymatically digested with collagenase/hyaluronidase (Stem Cell Technologies, cat#07919, Seattle, WA, USA) following the manufacturer’s protocol. Cells were then treated with 0.25% Trypsin/EDTA solution (Stem Cell Technologies, cat #07901, Seattle, WA, USA) and 5 mg/mL dispase (Gibco, cat#17105-041, Waltham, MA, USA).

### 2.4. Flow Cytometry and Cell Sorting

Details for flow cytometry and cell sorting are described in the Supplemental Materials and Methods (*Flow Cytometry and Cell Sorting*). Antibodies and dilutions used for cell staining are shown in Supplemental Table S2.

### 2.5. Mammosphere Culture and Limiting Dilution Assay

Details of mammosphere culture, limiting dilution assays, and media components are described in the Supplemental Materials and Methods (*Mammosphere Culture and Limiting Dilution Assay*).

### 2.6. Conditioned Medium (CM) Assay

Details for the CM assay are described in the Supplemental Materials and Methods. Recombinant Igf1 (Life Technologies, cat# PHG0071, Waltham, MA, USA) and picropodophyllin (PPP, Santa Cruz, cat# sc-204008, Santa Cruz, CA, USA) were added to the CM at a final concentration of 7.5 nM and 10 μM, respectively. DMSO vehicle was added as treatment control.

### 2.7. RT-qPCR and Western Blot Analysis

RT-qPCR and Western blot analysis were performed as described in the Supplemental Materials and Methods (*RNA Extraction and RT-qPCR* and *Western Blot Analysis*). Primer sequences for RT-qPCR are listed in Supplemental Table S3. Target gene expression was normalized to TATA-binding protein (TBP), and the 2^−ΔΔCt^ method was used to calculate relative gene expression levels. Antibodies and dilutions for Western blot analysis are listed in Supplemental Table S2. Signals of target protein bands were normalized to GAPDH bands of the same sample and then normalized to the control group to calculate fold changes.

### 2.8. ELISA Assay

Protein concentrations of Igf1 in CM were measured using the Mouse Igf1 ELISA Kit PicoKine™ (Boster Bio, cat #EK0378, Pleasanton, CA, USA), following the manufacture’s protocol.

### 2.9. DNA Methylation Analysis

Genomic DNA was extracted from mammary stromal cells using the DNeasy Blood & Tissue kit (Qiagen, cat #69504, Redwood City, CA, USA). Bisulfite conversion of genomic DNA was performed with a sodium bisulfite kit (EZ DNA Methylation-Lightning Kit, Zymo Research, cat #D5030, Irvine, CA, USA), following the manufacturer’s instructions. Bisulfite primers used to cover CG sites on Pr1 and Pr2 are listed in Supplemental Table S3. PCR conditions were 95 °C for 3 min, 95 °C for 15 s, annealing at 55 °C for 20 s, 72 °C for 20 s, 38 cycles. PCR products were cloned into the T-vector pMD19 (TAKARA Bio Inc., cat#3271, San Jose, CA, USA). At least ten clones for each sample were sequenced with M13 forward or reverse primers (Eton Bioscience Inc., San Diego, CA, USA). DNA methylation analysis was performed using the quantification tool for methylation analysis (QUMA, RRID:SCR_010907) [23].

### 2.10. siRNA Transfection

NIH 3T3 cells were grown to 70% confluency in DMEM/Ham’s F-12 (Caisson Labs, cat#DFP17, Smithfield, VA, USA) culture media with addition of 10% fetal bovine serum (FBS). Cells were trypsinized and replated in 12-well plates. After reaching 50% confluency, cells were treated with Lipofectamine RNAiMAX transfection reagent (Invitrogen, cat #13778100, Waltham, MA, USA) with addition of 20 nM *Dnmt3b*-specific siRNA (ThermoFisher, cat #161533, Waltham, MA, USA) or non-targeting control siRNA (ThermoFisher, cat # AM4611, Waltham, MA, USA), according to the manufacturer’s protocols. Cells were then maintained in DMEM/Ham’s F-12 culture media with addition of 2% FBS for 48 h before collection.

### 2.11. Carcinogen Treatment

Starting at 7 to 9 weeks of age, a separate group of diet-exposed mice (*n* = 17/HT-A; *n* = 33/HT-B; *n* = 23/LT-C; *n* = 17/LT-D) were administered daily low doses (20 μg) of 7, 12 dimethylbenz[a]anthracene (DMBA) by oral gavage, 5 days per week for 6 weeks, as described previously [24]. Animals were checked daily for health status and tumors palpated on a daily basis to monitor size and growth.

### 2.12. Histological Analyses and Immunohistochemistry

Preparation of histological sections and staining were performed by the CVMBS Research Histology Unit at Texas A&M University. Tumors were fixed in formalin and slides were stained with hematoxylin and eosin and were classified by a trained pathologist using the classification systems proposed by Cardiff et al. [25]. The pathologist was blinded to descriptive information about the tumor, including size, animal group, time of detection. Twenty-two tumors (*n* = 11/HT-A; *n* = 11/HT-B) from HT animals and eleven tumors from LT animals (*n* = 7/LT-C; *n* = 4/LT-D) were analyzed to determine histological subtypes. Details of immunohistochemical procedures are described in the Supplemental Materials and Methods (*Immunohistochemistry Staining*), and antibodies and dilutions are shown in Supplemental Table S2.

### 2.13. Statistics

Data were tested for the normal distribution using the Shapiro–Wilk normality test. Statistical differences between two groups were determined using Student’s *t*-test and among more than two groups using ANOVA with Tukey’s multiple comparison post-test, unless otherwise specified. Numerical results reflect mean ± SEM. Correlation analysis for DNA methylation and gene expression was performed using Pearson’s correlation test. To assess the frequency of mammosphere-initiating cells, an extreme limiting dilution analysis (ELDA) was performed as described previously [26,27], and pairwise differences between the groups were compared with likelihood ratio tests using the asymptotic chi-squared test approximation to the log-ratio [26,27]. Tumor latency was compared with the Kaplan–Meier method, and statistical significance was determined with the log-rank test. *p* < 0.05 was considered significant.

ELDA analysis was performed using the ELDA web tool [27]. All the other statistical analyses were conducted, and graphical representations of data were plotted using the GraphPad Prism 8 software (RRID:SCR_002798).

## 3. Results

### 3.1. Mammary Glands from Pre- and Post-Pubertal Female Mice in the HT-A and HT-B Groups Had an Expanded MaSC-Enriched Compartment

**Figure 2:**
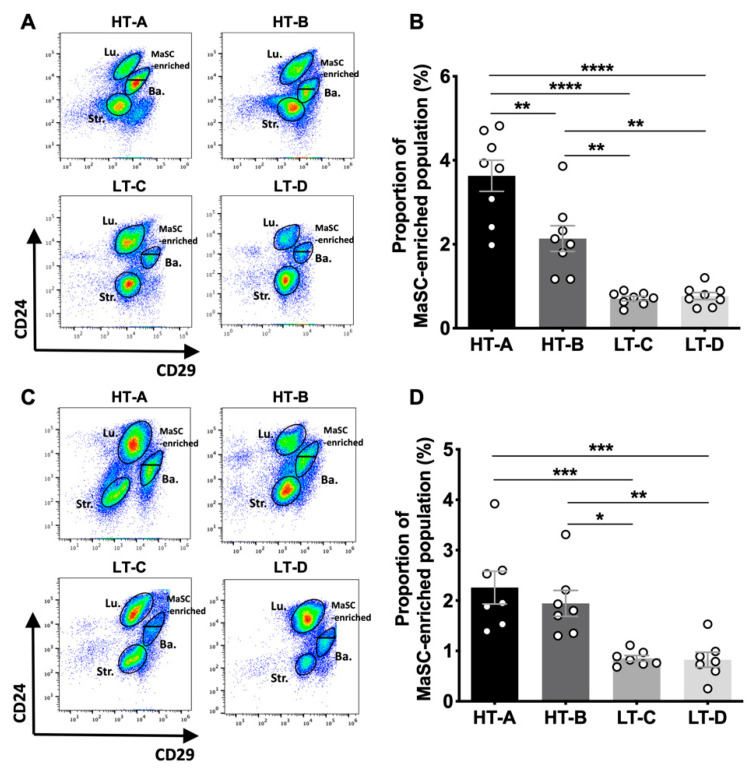
The HT-A and HT-B dietary regimens expanded the MaSC-enriched compartment in both pre- and post-pubertal female mice. (**A**) Representative gating plots of the MaSC-enriched population in 5-week-old mammary glands. MaSC-enriched, basal (Ba.), luminal (Lu.), and stromal (Str.) cells were gated into Lin^-^CD24^med^CD29^hi^, Lin^−^CD24^+^ CD29^hi^, Lin^−^CD24^hi^CD29^low^, and Lin^-^CD24^low^CD29^low^ subpopulations, respectively; (**B**) Frequency of MaSC-enriched populations in mammary glands from 5-week-old animals (*n* = 8/group); (**C**) Representative gating plots of the MaSC-enriched population in 10-week-old mammary glands; (**D**) Frequency of MaSC-enriched populations in mammary glands from 10-week-old animals (*n* = 7/group). Circles in the bar graphs represent individual data points collected from independent experiments. Mean ± SEM is shown. Differences were compared by one-way ANOVA and pairwise comparisons were performed using the Tukey’s posttest. * *p* < 0.05, ** *p* < 0.01, *** *p* < 0.001, **** *p* < 0.0001.

Since we aimed to investigate the effect of early dietary exposures on the mammary gland, we collected tissues from 5-week and 10-week-old mice that were not treated with carcinogen (Figure 1). To assess the effect of the dietary regimens on mammary stem cell (MaSC) numbers, we performed fluorescence-activated cell sorting (FACS) analyses of mammary epithelial cells (MECs). The MaSC-enriched population was identified by previously validated cell surface markers (Lin^−^/CD24^med^/CD29^hi^) [28,29] and the gating strategy is shown in Supplemental Figure S1A. In mammary tissues from prepubertal mice, the MaSC-enriched population from group HT-A was 3.5-times higher than in groups LT-C and LT-D (Figure 2A,B). Similarly, the MaSC-enriched population from group HT-B was 2.6 times higher than both LT groups (Figure 2A,B). In postpubertal animals, the MaSC-enriched population from both HT groups was still increased by 2.0 times compared to the LT groups (Figure 2C,D). These results indicate that exposure to the HT-A and HT-B dietary regimens expanded the MaSC-enriched compartment before puberty, and this expansion was maintained postpubertally.

The analysis of the total number of MECs dissociated from abdominal/inguinal mammary glands revealed that prepubertal animals from group HT-A had twice the number of MECs compared to age-matched LT groups (Supplemental Figure S1B). When multiplying the frequency of the MaSC-enriched population by the total number of MECs, the absolute number of cells with MaSC markers in prepubertal HT-A animals was 4.2 times higher than in age-matched LT groups (Supplemental Figure S1C). In postpubertal glands, the number of MECs from both HT groups was 2.0 times higher than the LT animals. Therefore, the absolute number of MaSCs was 3.7 times higher in HT compared to age-matched LT animals (Supplemental Figure S1D,E). These results demonstrate that the total number of carcinogen-susceptible cell targets was significantly higher in mammary tissues from mice in HT, compared to LT diet groups.

### 3.2. The Number of Mammosphere-Initiating Cells Was Increased in Mice from the HT Dietary Regimen Groups (HT-A and HT-B)

Due to the lack of an exclusive marker for isolating MaSCs and the compositional heterogeneity of the sorted Lin^-^/CD24^med^/CD29^hi^ population, MaSCs cannot be directly enumerated by FACS [28,29]. An intrinsic characteristic of MaSCs is their ability to self-renew and form sphere-like colonies (mammospheres) in non-adherent cultures [30]. The MaSC frequency in a given population of MECs is reflected by the number of mammospheres formed, particularly in the second and subsequent serial passages [26,31]. We compared the mammosphere-forming efficiency of second passage (P2) MECs from mammary glands from mice in HT and LT groups and performed a mammosphere Limiting Dilution Assay (LDA) of third passage (P3) MECs to reliably quantitate MaSC frequency [26,27].

**Figure 3:**
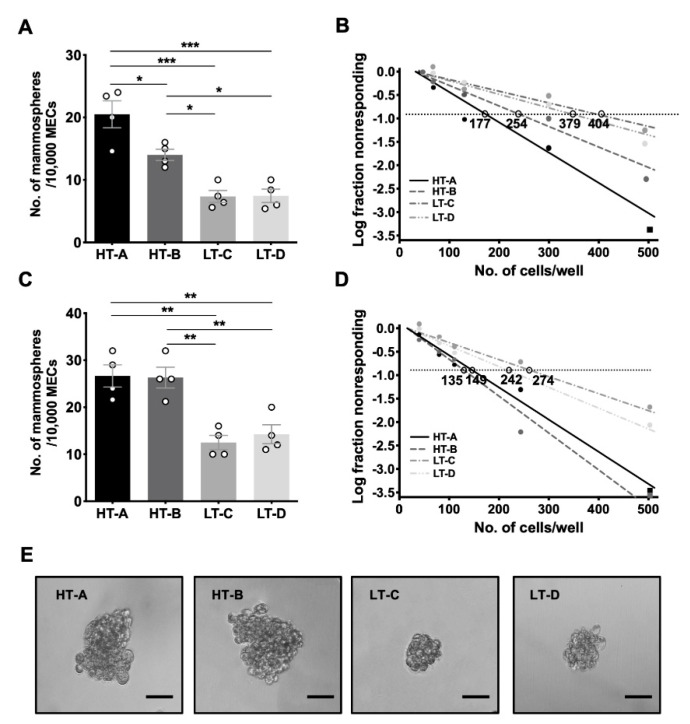
The HT-A and HT-B dietary regimens increased the number of mammosphere−initiating cells. (**A**) P2 mammospheres formed by MECs from 5−week−old prepubertal animals. Four independent experiments (*n* = 4/group) were performed and mean ± SEM are shown; (**B**) Limiting Dilution Assays (LDAs) of prepubertal P3 mammospheres. The natural log fraction of non-responding wells plotted on a linear scale versus the cell density per well; (**C**) P2 mammospheres formed by MECs from 10-week-old postpubertal animals. Four independent experiments (*n* = 4/group) were performed; (**D**) LDAs of postpubertal P3 mammospheres. The natural log fraction of non-responding wells plotted on a linear scale versus the cell density per well; (**E**) Representative images of postpubertal mammospheres. Scale bar, 50 µm. P2 mammospheres were compared with one-way ANOVA and Tukey’s posttest. * *p* < 0.05, ** *p* < 0.01, *** *p* < 0.001.

In 5-week-old prepubertal animals, P2 MECs from mice in the HT groups formed significantly more mammospheres than those from the LT groups (Figure 3A). The LDA (Figure 3B and Supplemental Table S4) revealed that the frequency of MaSCs in glands from HT-A mice was approximately 2 times higher than age-matched animals from group LT-C (1 in 177 vs. 1 in 404) or LT-D (1 in 177 vs. 1 in 379). Similarly, the frequency of MaSCs in glands from HT-B animals was 1.5 times higher than in LT-C (1 in 254 vs. 1 in 404) or LT-D animals (1 in 254 vs. 1 in 379).

In 10-week-old postpubertal animals, the number of P2 mammospheres formed by HT MECs was also significantly increased, compared to either LT group (Figure 3C). The LDA (Figure 3D and Supplemental Table S5) revealed that the frequency of MaSCs in glands from HT-A and HT-B animals was 1.8 times higher than in LT groups (avg. 1 in 143 vs. 1 in 258). Furthermore, we observed that mammosphere sizes formed by postpubertal MECs from both HT groups were larger than those from both LT groups (average 100μM vs. average 50μM in diameter), indicating a higher proliferative capacity of the HT progenitor cells (Figure 3E). Taken together, the FACS and mammosphere assay results demonstrated that the HT-A and HT-B dietary regimens stimulated a significant increase in MaSCs compared to the LT-C and LT-D diets.

### 3.3. The HT-A and HT-B Dietary Regimens Increased Mammary Igf1 Levels and Decreased Igfbp5 Levels

A recent human study shows that increased circulating Igf1 levels are associated with increased breast cancer risk in a large cohort (*n* = 206, 263) of women [32]. Our study in the BK5.Igf1 transgenic mouse model show that Igf1 overexpression in keratin 5 (K5)-positive mammary basal epithelial cells promotes the symmetric division of MaSCs and increases MaSC numbers in vivo [33]. In addition, we found in our previous study with the crossover-feeding model that serum Igf1 levels in 5-month-old animals from the HT susceptible groups are significantly higher than the age-matched LT groups [8]. Based on this evidence, we hypothesized that the HT-A and HT-B dietary regimens increase the number of MaSCs by inducing elevated levels of Igf1.

**Figure 4:**
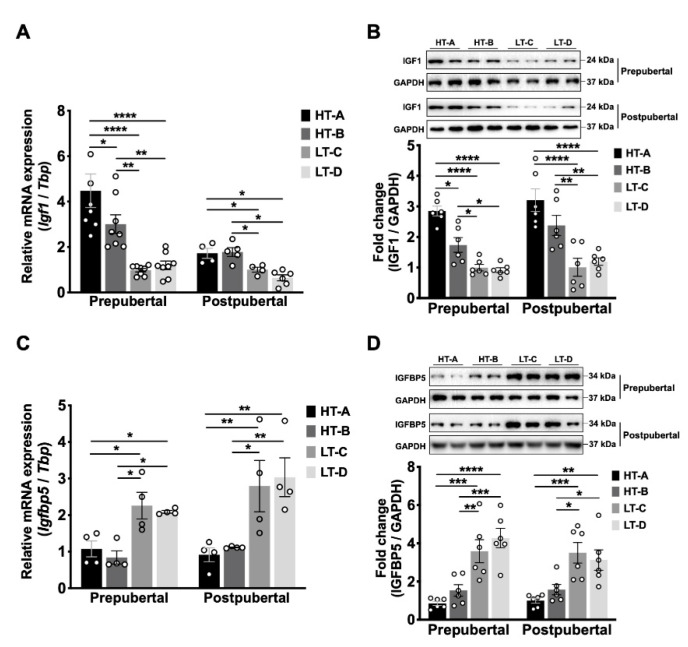
The HT-A and HT-B dietary regimens increased mammary Igf1 levels and decreased Igfbp5 levels. (**A**) Mammary tissue *Igf1* mRNA levels of 5-week-old (*n* = 8/group) and 10-week-old (*n* = 5/group) animals. Relative *Igf1* mRNA levels are expressed as fold change over group LT-C; (**B**) Igf1 protein levels in 5-week-old and 10-week-old (*n* = 6/group) mammary tissues. Top panel, representative Western blot, bottom panel, densitometric quantification; (**C**) *Igfbp5* mRNA levels in 5-week-old and 10-week-old (*n* = 4/group) mammary tissues. Relative mRNA levels are expressed as fold change over group HT-A; (**D**) Igfbp5 protein levels in 5-week-old and 10-week-old (*n* = 6/group) mammary tissues. Top panel, representative Western blot, bottom panel, densitometric quantification. Circles in the bar graphs represent individual data points collected from independent experiments. Mean ± SEM is shown. One-way ANOVA was used for statistical analysis, and pairwise comparisons were performed using Tukey’s posttest. * *p* < 0.05, ** *p* < 0.01, *** *p* < 0.001, **** *p* < 0.0001.

We first tested this hypothesis by measuring mammary tissue Igf1 levels in both 5-week-old (prepubertal) and 10-week-old (postpubertal) female mice. Prepubertal mammary glands from group HT-A expressed significantly higher *Igf1* mRNA levels than both LT groups (Figure 4A), and there was a corresponding, highly significant, increase in Igf1 protein levels (Figure 4B). Similarly, prepubertal HT-B tissues also expressed significantly higher *Igf1* mRNA and protein levels than both LT groups (Figure 4A,B). *Igf1* mRNA and protein levels in group HT-A were higher than in HT-B (Figure 4A,B), likely due to the longer exposure time to the HFHS diet of the HT-A animals, which were exposed during both lactation and postweaning. In postpubertal glands, the tissue levels of *Igf1* mRNA and protein were also significantly increased in HT compared to LT groups (Figure 4A,B). These results show that exposure to the HT-A and HT-B dietary regimens increased mammary tissue Igf1 levels prepubertally, and this increase persisted into adulthood.

The bioavailability of Igf1 in the mammary gland is regulated by Igf-binding proteins (Igfbps) [34,35]. Igfbps consist of six protein members (Igfbp1-6), of which Igfbp5 has been shown to play critical roles in regulating the proliferation, apoptosis, and differentiation of MECs [34]. Igfbps can inhibit Igf1 signaling by sequestering free Igf1, reducing its binding to the Igf1 receptor (Igf1r) [35]. Our data show that *Igfbp5* mRNA and protein levels were significantly decreased in mammary glands from HT compared to LT groups at both pre- and postpubertal stages (Figure 4C,D). These findings indicate that the HT-A and HT-B dietary regimens not only increased total mammary Igf1 expression levels, but also increased Igf1 bioavailability through the downregulation of Igfbp5.

### 3.4. The HT-A and HT-B Dietary Regimens Increased Igf1 Production in Mammary Stromal Cells and Promoted MaSC Self-Renewal

We next investigated whether the differences in tissue Igf1 levels in HT- and LT animals affected the self-renewal capacity of MaSCs. Previous studies show that before 8 weeks of age, *Igf1* mRNA is expressed in both mammary ductal epithelium and stroma in virgin female mice. After 8 weeks, *Igf1* expression is restricted to mammary stroma [36].

**Figure 5:**
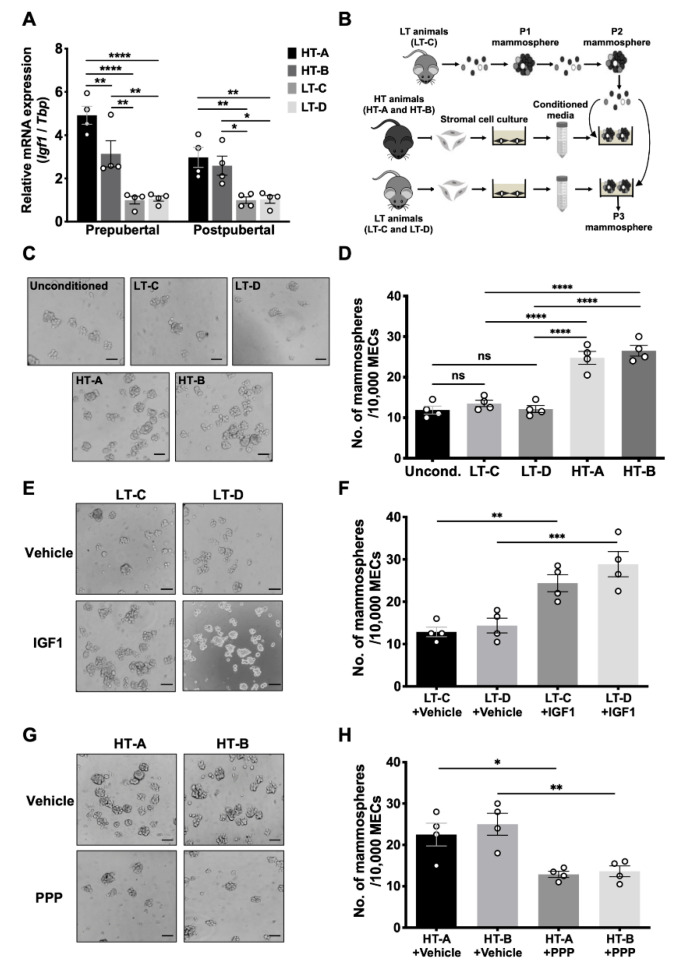
The HT-A and HT-B dietary regimens increased Igf1 production in mammary stromal cells and promoted MaSC self-renewal. (**A**) *Igf1* mRNA levels in FACS-sorted mammary stromal cells from 5-week-old and 10-week-old tissues from the HT and LT groups (*n* = 4/group). Relative *Igf1* mRNA levels are expressed as fold change over group LT-C; (**B**) Experimental procedure of the mammosphere assay using stromal cell-conditioned medium (CM); (**C**) Representative images and (**D**) bar graph showing numbers of mammospheres formed in CM produced by HT and LT groups. Unconditioned DMEM/Ham’s F-12 medium was used as a control; (**E**) Representative images and (**F**) bar graph showing numbers of mammospheres formed in LT-C-CM and LT-D-CM, supplemented with DMSO vehicle or 7.5 nM recombinant Igf1; (**G**) Representative images; (**H**) Bar graph showing numbers of mammospheres formed in HT-A-CM and HT-B-CM, supplemented with DMSO vehicle or 10 μM picropodophyllin (PPP). Scale bar of images = 100 µm. Four independent experiments (*n* = 4) were performed, and circles in the bar graphs represent individual data points collected from independent experiments. Mean ± SEM is shown. One-way ANOVA was used for statistical analysis, and pairwise comparisons were performed using Tukey’s posttest. * *p* < 0.05, ** *p* < 0.01, *** *p* < 0.001, **** *p* < 0.0001; ns, not significant.

We sorted the MaSC-enriched population (Lin^-^CD24^med^CD29^hi^), luminal epithelial cells (Lin^-^CD24^hi^CD29^low^), and stromal cells (Lin^-^CD24^low^CD29^low^) from mammary tissues by FACS (Supplemental Figure S1A). *Igf1* mRNA in MaSC-enriched and luminal epithelial cells from 5-week-old prepubertal animals was not differentially expressed in HT versus the LT groups (Supplemental Figure S2A,B). However, *Igf1* mRNA levels in mammary stromal cells were 5.6 times higher in group HT-A and 3.7 times higher in group HT-B, compared to average of the LT animals (Figure 5A). In 10-week-old postpubertal animals, *Igf1* mRNA levels were 2.9 times higher in mammary stromal cells from HT compared to the LT groups (Figure 5A).

To assess whether this increased mammary stromal *Igf1* expression affected the ability of MaSCs to self-renew, we performed a mammosphere-forming assay using medium conditioned by mammary stromal cells derived from either HT or LT- tissues (Figure 5B). Briefly, FACS-sorted mammary stromal cells harvested from prepubertal HT and LT groups were incubated separately in serum-free, stromal cell growth medium, and the supernatant (conditioned medium, CM) was collected. At the same time, prepubertal MECs from group LT-C were harvested and cultured to generate P2 mammospheres, as described above. P2 mammospheres were then again disaggregated and re-plated using the previously harvested CM. Cells were incubated in their respective CM for seven days to form P3 mammospheres.

As shown in Figure 5C,D, CM produced by group LT-C (LT-C-CM) or LT-D (LT-D-CM) did not significantly affect mammosphere-forming efficiency compared to unconditioned mammosphere growth medium. Conversely, the number of mammospheres was significantly increased in HT-A-CM or HT-B-CM, compared to LT-C-CM and LT-D-CM (Figure 5C,D). These results indicate that mammary stromal cells from the HT-susceptible groups secreted soluble factors that were able to promote the self-renewal capacity of MaSCs derived from a group not exposed to the pro-tumorigenic diet regimens.

We then conducted a series of experiments to demonstrate that Igf1 was the soluble factor responsible for these effects. First, ELISA data confirmed that Igf1 protein concentrations were increased by 3.2 times in CM generated by the HT groups, compared to the LT groups (avg. 0.29 nM vs. avg. 0.09 nM) (Supplemental Figure S2C). Second, the addition of recombinant Igf1 to LT-C-CM or LT-D-CM significantly increased the number of mammospheres formed, recapitulating the proliferative effects of HT-A-CM or HT-B-CM (Figure 5E,F and Supplemental Figure S2D). Third, we added the Igf1r inhibitor picropodophyllin (PPP) to CM-treated cultures. When PPP was added to cultures treated with HT-A-CM or HT-B-CM, mammosphere numbers were significantly decreased compared to cultures without PPP (Figure 5G,H and Supplemental Figure S2E). However, PPP did not affect the number of mammospheres formed in unconditioned growth medium (Supplemental Figure S2E) or CM from the LT groups (Supplemental Figure S2F–G), indicating that the inhibitory effect of PPP on sphere formation in HT-A-CM or HT-B-CM was not due to non-specific cytotoxicity. These results demonstrate that this increase in mammosphere numbers observed in cultures treated with HT-A-CM or HT-B-CM was due to increased Igf1 levels and the activation of Igf1r signaling.

### 3.5. The HT-A and HT-B Dietary Regimens Decreased DNA Methylation of the Igf1 Pr1 Promoter in Mammary Stromal Cells

**Figure 6:**
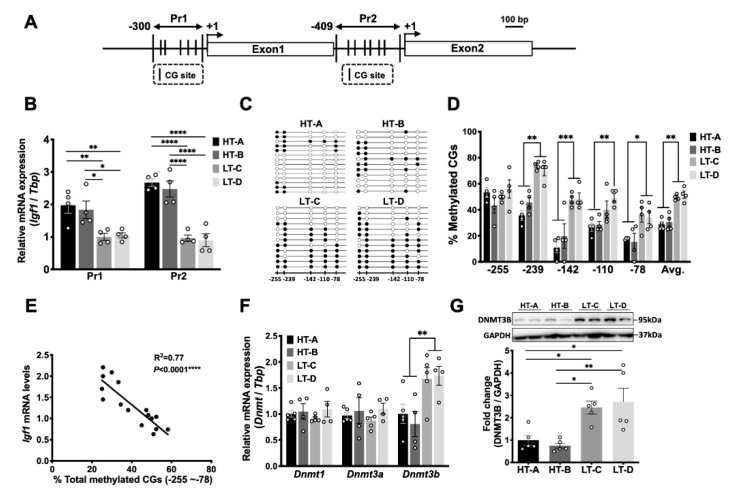
The HT-A and HT-B dietary regimens decreased DNA methylation of the *Igf1* Pr1 promoter in mammary stromal cells, and Dnmt3b levels were also decreased. (**A**) Graphical representation of the mouse *Igf1* promoters 1 (Pr1) and 2 (Pr2). Transcription start sites (TSSs, +1) are shown as arrows, CG sites are shown as vertical bars; (**B**) mRNA levels of Pr1 and Pr2 transcripts in mammary stromal cells from 5-week-old animals (*n* = 4/group). Relative mRNA levels are expressed as fold change over group LT-C; (**C**) Representative Pr1 DNA methylation patterns from each group. Each circle represents a CG site; closed circles are methylated. Each row indicates sequencing results of a single clone; (**D**) Quantification of DNA methylation percentages for each CG site of the Pr1 promoter in mammary stromal cells from 5-week-old animals (*n* = 4/group); (**E**) Scatter plot of Pr1 mRNA levels and combined Pr1 methylation percentages (Pearson’s correlation test, R^2^ = 0.77, *p* < 0.0001, one-tailed); (**F**) mRNA levels of DNA methyltransferases in sorted mammary stromal cells from 5-week-old animals (*n* = 4/group). Relative mRNA levels are expressed as fold change over group HT-A; (**G**) Dnmt3b protein levels in stromal cells. Top panel, representative Western blot, bottom panel, densitometric quantification (*n* = 5/group). Circles in the bar graphs represent individual data points collected from independent experiments. Mean ± SEM is shown. One-way ANOVA was used for statistical analysis and pairwise comparisons were performed using Tukey’s posttest. * *p* < 0.05, ** *p* < 0.01, *** *p* < 0.001, **** *p* < 0.0001.

*Igf1* transcription is driven by two alternative promoters in both humans and mice (Figure 6A). Promoter 1 (Pr1) initiates transcription from exon 1, while promoter 2 (Pr2) initiates from exon 2 [37]. In rodents, Pr1 becomes active during the embryonic stage and remains active through adulthood, whereas Pr2 remains silent until 3 weeks of age [37]. Both transcript isoforms generate the same mature Igf1 peptide [37].

The QPCR analysis of FACS-sorted mammary stromal cells showed that both Pr1 and Pr2 transcripts were expressed at significantly higher levels in 5-week-old, prepubertal mice in HT groups compared to age-matched LT animals (Figure 6B). To examine whether this increase in *Igf1* transcription was due to hypomethylation of one or both promoters, we performed bisulfite sequencing analyses to study methylation patterns of the CG sites that flank the Pr1 and Pr2 promoter transcription start sites (TSS) (Figure 6A). Figure 6C,D show that Pr1 was hypomethylated in mammary stromal cells from prepubertal HT animals on four out of five CG sites, compared to the LT animals. Of the CG sites, 41.0% of CG-239 were methylated in the HT groups, compared to 72.0% of CG-239 in the LT groups; 15.5% of CG-142 in HT groups vs. 48.5% of CG-142 in LT groups; 27.5% of CG-110 in HT groups vs. 44.5% of CG-110 in LT groups; 16.5% of CG-78 in HT groups vs. 35% of CG-78 in LT groups (Figure 6D). *Igf1* Pr1 mRNA levels in each stromal cell sample were inversely correlated with the total DNA methylation percentages of the five Pr1 CG sites (Figure 6E, R^2^ = 0.77, *p* < 0.0001). These results indicate that the HT-A and HT-B dietary regimens increased *Igf1* expression in mammary stromal cells by inducing DNA hypomethylation of the Pr1 promoter.

Since Pr2 transcripts were also significantly upregulated in HT-susceptible groups compared to the LT groups (Figure 6B), we analyzed DNA methylation levels on six CG sites (CG-371-CG-255) that flank the Pr2 TSS (Figure 6A). However, these CG sites were largely unmethylated in both HT and LT groups (Supplemental Figure S3A,B), indicating that DNA methylation of distal enhancers, or other epigenetic mechanisms, such as histone modifications, likely contributed to this differential expression of the Pr2 transcript in these animals.

Genomic DNA methylation is catalyzed by DNA methyltransferases (DNMTs), including DNMT1, DNMT3A and Dnmt3b. Gene expression levels of *Dnmts* were measured in FACS-sorted mammary stromal cells, showing that *Dnmt3b* mRNA levels were significantly lower in 5-week-old HT animals compared to the LT groups, while gene expression levels of *Dnmt1* and *Dnmt3a* were not different (Figure 6F). Western blot analysis of mammary stromal cells demonstrated that Dnmt3b protein levels were also significantly decreased in the HT groups compared to the LT groups (Figure 6G). These results suggest that the diet-induced hypomethylation of Pr1 was mediated by decreased Dnmt3b levels.

### 3.6. Dnmt3b Knockdown Decreased Igf1 Pr1 DNA Methylation and Increased Igf1 mRNA Expression

**Figure 7:**
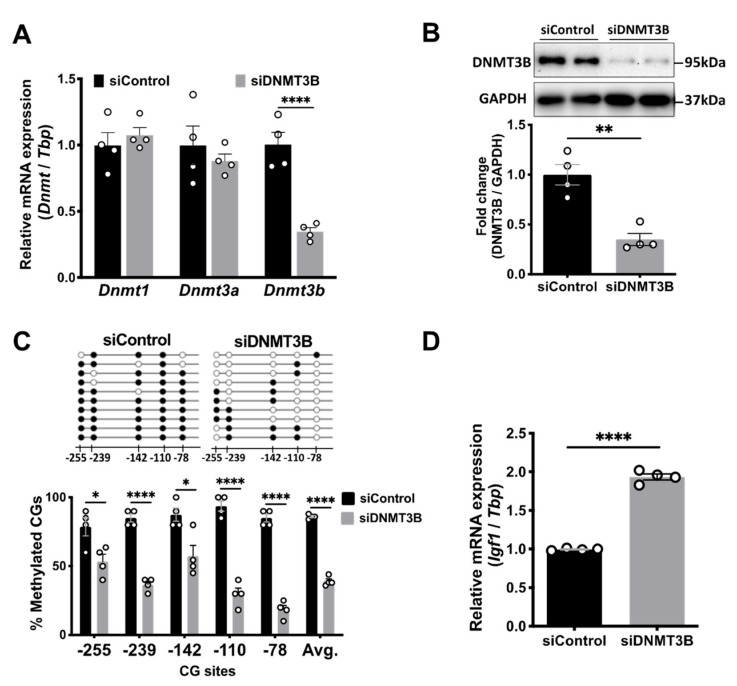
*Dnmt3b* knockdown decreased *Igf1* Pr1 DNA methylation and increased *Igf1* mRNA expression. (**A**) mRNA levels of *Dnmts* in 3T3 cells transfected with non-targeting siRNA or *Dnmt3b*-specific siRNA. Relative mRNA levels are expressed as fold change over non−targeting control. Four independent experiments (*n* = 4) were performed; (**B**) Dnmt3b protein levels in non-targeting siRNA- and *Dnmt3b* siRNA−treated groups. Top panel, representative Western blot, bottom panel, densitometric quantification of four independent experiments; (**C**) Representative *Igf1* Pr1 DNA methylation patterns (top panel) and quantification of methylated CG sites in four independent experiments (bottom panel); (**D**) *Igf1* mRNA levels in transfected 3T3 cells. Four independent experiments (*n* = 4) were performed, and circles in the bar graphs represent individual data points collected from independent experiments. Mean ± SEM is shown. Pairwise comparisons were performed using Student’s *t*-test. * *p* < 0.05, ** *p* < 0.01, **** *p* < 0.0001.

We then investigated whether there was a direct causal relationship between DNA hypomethylation of the *Igf1* Pr1 promoter and decreased Dnmt3b levels in vitro. Since *Igf1* is primarily expressed in mammary stromal fibroblasts [38], we knocked down *Dnmt3b* by transfecting *Dnmt3b* siRNA into NIH 3T3 cells, a mouse embryonic fibroblast cell line. *Dnmt3b* siRNA transfection reduced *Dnmt3b* mRNA levels by 60% after 48 h compared to the non-targeting siRNA control (Figure 7A). In contrast, the mRNA levels of *Dnmt1* and *Dnmt3a* were not significantly changed, indicating a high specificity of the *Dnmt3b* knockdown (Figure 7A). Dnmt3b protein levels were also decreased by 65% in siRNA-treated cells compared to non-targeting controls (Figure 7B).

To assess whether this *Dnmt3b* knockdown resulted in *Igf1* Pr1 hypomethylation, we performed bisulfite sequencing analyses of *Igf1* Pr1 in these *Dnmt3b* siRNA-transfected cells. As shown in Figure 7C, *Dnmt3b* knockdown significantly decreased *Igf1* Pr1 methylation of all five CG sites that flank the TSS. Concordant with this, *Igf1* mRNA levels were significantly increased in *Dnmt3b* siRNA-transfected cells compared to the non-targeting siRNA controls (Figure 7D). These results show that *Dnmt3b* knockdown induced *Igf1* Pr1 hypomethylation, resulting in an increase in *Igf1* gene expression.

### 3.7. The HT Groups Developed Increased Proportions of Metaplastic Carcinomas

**Figure 8:**
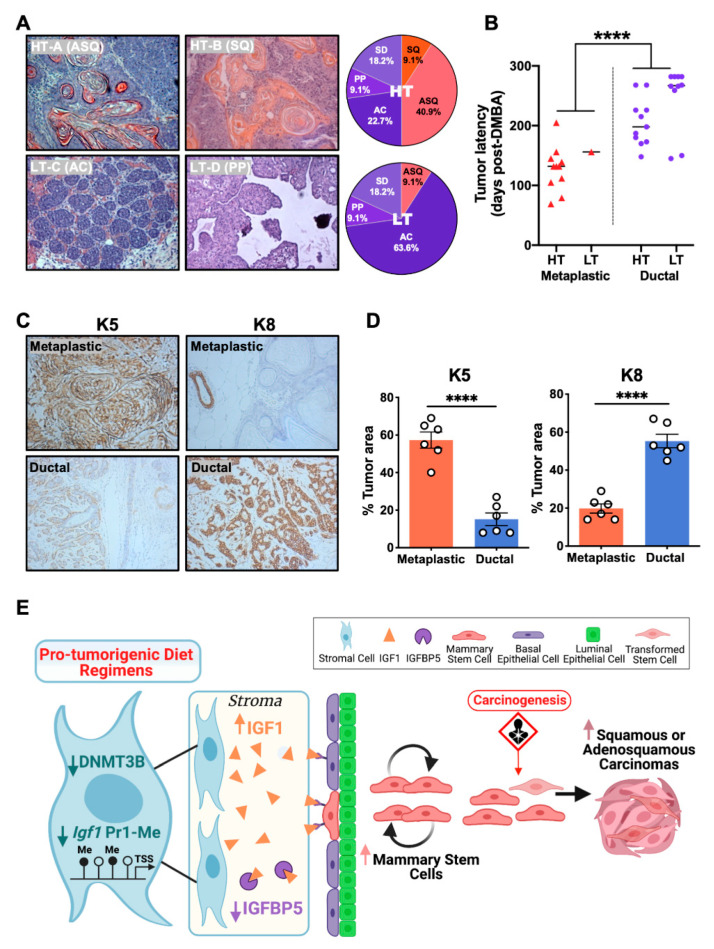
DMBA-treated animals receiving the HT-A and HT-B dietary regimens developed increased proportions of metaplastic carcinomas. (**A**) Representative hematoxylin and eosin images (magnification = 10X) and distribution of tumor histopathological types in the HT (*n* = 22) and LT (*n* = 11) groups. ASQ, adenosquamous carcinoma; SQ, squamous carcinoma; AC, acinar carcinoma; PP, papillary carcinoma; SD, solid carcinoma; (**B**) Tumor latency of metaplastic carcinomas (*n* = 12) and ductal adenocarcinomas (*n* = 21) in the HT and LT groups. Latency analysis was performed with the Kaplan–Meier method, and statistical significance was determined with the log-rank test. **** *p* < 0.0001; (**C**) Representative images of immunohistochemical staining of metaplastic carcinomas and ductal adenocarcinomas. Left panel, K5; right panel, K8 staining; (**D**) Quantification of K5 (left panel) and K8 (right panel) expression in mammary tumors (*n* = 7). Student’s *t*-test, **** *p* < 0.0001; (**E**) Proposed model of mechanisms by which the HT-A and HT-B dietary regimens increase mammary tumor susceptibility.

To investigate the effect of the HT-A and HT-B dietary regimens on the development of mammary tumor subtypes, a separate group of diet-exposed animals were treated with DMBA starting at 7 to 9 weeks of age, as described previously [24]. The histological sections of mammary tumors were analyzed and classified by phenotype. Twenty-two tumors from the HT groups (eleven from HT-A and eleven from HT-B) and eleven tumors from the LT groups (seven from LT-C and four from LT-D) were analyzed (Supplemental Table S6). In the HT groups, eleven tumors (50.0%) were classified as metaplastic carcinomas of the squamous or adenosquamous phenotype, and these tumors were characterized by squamous metaplasia with widespread keratin pearls (Figure 8A). Eleven tumors (50.0%) were classified as ductal adenocarcinomas of either the acinar, papillary, or solid type, typically with central necrosis (Supplemental Table S6). Conversely, in the LT groups, only one tumor (9.1%) was classified as an adenosquamous carcinoma, while the majority of tumors (90.9%) were ductal adenocarcinomas of the acinar, papillary, or solid phenotype (Figure 8A and Supplemental Table S6). The overall proportion of metaplastic carcinomas was significantly higher in HT than in LT groups (Figure 8A, chi-square test, *p* = 0.02). In addition, the latency of metaplastic carcinomas was significantly shorter than for ductal adenocarcinomas (Figure 8B).

We next quantified expression levels of the basal (keratin 5, K5) and luminal (keratin 8, K8) lineage markers [15] in the histological tumor sections. The immunohistochemical analysis of the metaplastic carcinomas from the HT groups revealed a significantly higher expression of K5 and lower expression of K8, when compared to the ductal adenocarcinomas from the LT groups (Figure 8C,D). These results demonstrated that mice fed the HT-A and HT-B dietary regimens were more likely to develop metaplastic carcinomas, indicating that dietary manipulations can affect mammary tumor subtypes resulting from carcinogen treatment.

## 4. Discussion

This study showed how early dietary manipulations during the gestational, lactational and/or postweaning time periods program mammary cancer risk later in life. Mechanistically, this occurred through an increased and sustained activation of the Igf1 signaling pathway, resulting in a responsive increase in the number of carcinogen-susceptible MaSCs. Based on our data, we propose the following model (Figure 8E): the pro-tumorigenic HT-A and HT-B dietary regimens decreased Dnmt3b levels in mammary stromal cells, causing the hypomethylation of the *Igf1* Pr1 promoter, resulting in increased *Igf1* expression in this cell compartment. The HT-A and HT-B dietary regimens also decreased Igfbp5 levels in mammary tissues, resulting in an increase in Igf1 bioavailability. Elevated Igf1 levels promoted MaSC self-renewal, expanding the MaSC population, thereby increasing the number of carcinogen-susceptible cell targets, ultimately resulting in increased mammary carcinogenesis.

Carcinogen exposure of mice fed the HT-A and HT-B dietary regimens stimulated an increased formation of metaplastic (squamous and adenosquamous) mammary carcinomas, compared to animals exposed to the protective LT-C and LT-D diets, and metaplastic carcinomas displayed a shorter latency than the ductal adenocarcinomas. In humans, metaplastic carcinomas show the genomic and immunohistochemical features of basal-like breast cancers, which are characterized by aggressiveness, poor prognosis, and relatively high mortality [39,40]. Mammary squamous carcinomas in mice and basal-like breast cancers in humans display similarities in gene expression features and tend to be K5 positive, suggesting that mouse squamous tumors are basal-like [20]. In addition, recent comparative molecular studies demonstrate that the transcriptomic profiles of mouse metaplastic carcinomas overlap with the gene expression features of MaSCs [20,41]. Our data showed that exposure to the HT-A and HT-B diet regimens expanded the MaSC population, followed by a corresponding increase in the incidence of metaplastic carcinomas, suggesting that the metaplastic tumors in HT animals may have originated from transformed MaSCs.

Our data showed that the *Igf1* Pr1 promoter was hypomethylated in mice exposed to the HT-A and HT-B dietary regimens. The nucleotide sequences of the *Igf1* promoters are highly conserved between humans and rodents (85–92% sequence similarity) [42], and the differential methylation of *Igf1* promoters has also been reported in human placenta and in peripheral blood mononucleocytes [43,44]. Therefore, the epigenetic alterations of the *Igf1* Pr1 in HT diet-exposed mice likely recapitulate a similar effect occurring in humans consuming a high-fat, high-sugar, ‘Western’ diet that begins in infancy or childhood. Since this epigenetic alteration of the *Igf1* Pr1 was observed in mammary stromal cells from prepubertal animals, and DNA methylation is a stable epigenetic marker that can be inherited through multiple cell divisions [13,45], our results also indicate that methylation of the *Igf1* Pr1 promoter in mammary tissues could be used as an epigenetic biomarker for early breast cancer risk assessment.

Emerging evidence shows that mammary stem and progenitor cells are potential tumor-initiating cells [17,18,19], and the lifetime risk for developing breast cancer is partly determined by the number of these cells in the mammary gland [8,16]. Hao et al. reported that circulating levels of adipose fatty acid binding protein (A-FABP) are significantly increased in obese patients with breast cancer compared to those without breast cancer, and in vitro stem cell sphere formation assays showed that A-FABP was able to promote sphere formation of both MCF-7 and E0771 mammary cancer cell lines [46]. In our animal model, increased stromal *Igf1* expression expanded the MaSC population in HT animals before puberty, thereby establishing increased mammary cancer risk during the early stages of mammary gland development. These results indicate that high-fat dietary exposures programmed mammary cancer risk prepubertally and prior to the onset of obesity, demonstrating that early life dietary interventions may be an effective breast cancer prevention strategy.

In summary, our study shows that exposure to the pro-tumorigenic HT-A and HT-B dietary regimens caused specific epigenetic alterations that increased *Igf1* expression in mammary stromal cells before puberty, ultimately resulting in a higher number of carcinogen-susceptible cell targets. These results demonstrated that mammary cancer susceptibility can be programmed before puberty and that effective dietary intervention strategies may reduce breast cancer incidence.

## Data Availability

Raw data for all experiments are available upon requests.

## Supplementary Materials

The supplementary information can be downloaded at: https://www.mdpi.com/article/10.3390/cells11162558/s1, Table S1: Composition of the experimental diets; Table S2: Antibodies; Table S3: Primers; Table S4: LDA statistical analysis for prepubertal mammospheres; Table S5: LDA statistical analysis for postpubertal mammospheres; Table S6: Tumor phenotypes; Figure S1: The total number of MaSCs was increased in HT compared to LT animals. (A) Gating strategy for cell sorting. MaSC-enriched, basal (Ba.), luminal (Lu.) and stromal (Str.) cells were gated into Lin-CD24^med^CD29^hi^, Lin-CD24^+^CD29^hi^, Lin-CD24^hi^CD29^low^ and Lin-CD24^low^CD29^low^ subpopulations, respectively. (B) Total numbers of rudimentary MECs harvested from abdominal/inguinal mammary glands from 5-week-old animals (*n* = 7/group). (C) Estimates of the total number of cells with MaSC markers in 5-week-old mice (*n* = 7/group). The frequency of the MaSC-enriched population was multiplied by the number of MECs isolated from each corresponding sample. (D) Total numbers of rudimentary MECs harvested from abdominal/inguinal mammary glands from 10-week-old animals (*n* = 5/HT-A; *n* = 7/HT-B; *n* = 4/LT-C; *n* = 4/LT-D). (E) Estimates of the total number of cells with MaSC markers in 10-week-old mice (*n* = 5/HT-A; *n* = 7/HT-B; *n* = 4/LT-C; *n* = 4/LT-D). Mean ± SEM are shown. One-way ANOVA was used for statistical analysis, and pairwise comparisons were performed using Tukey’s posttest, a ≠ b ≠ c, *P* < 0.05; Figure S2: (A) *Igf1* mRNA levels in FACS-sorted MaSC-enriched population and (B) luminal epithelial cells from mammary tissues of 5-week-old animals (*n* = 4/group). Relative Igf1 mRNA levels are expressed as fold change over group LT-C. (C) Igf1 protein concentrations in CM from HT and LT groups. Unconditioned DMEM/Ham’s F-12 medium was used as a negative control. Four independent experiments (*n* = 4) were performed. (D) Dose-response analysis to determine the effect of recombinant Igf1 on mammosphere numbers in LT-C-CM. (E) Dose-response analysis to determine the effect of picropodophyllin (PPP) on mammosphere numbers in unconditioned medium or in HT-A-CM. (F) Representative images and (G) bar graph showing numbers of mammospheres formed in LT-C-CM or LT-D-CM, supplemented with DMSO vehicle or 10 μM PPP. Scale bar = 100 µm. Four independent experiments (*n* = 4) were performed, and data are displayed as mean ± SEM. One-way ANOVA was used for statistical analysis and pairwise comparisons were performed using Tukey’s posttest. a≠b, *P* < 0.05; Figure S3: *Igf1* Pr2 was not differentially methylated in mammary stromal cells from HT and LT groups. (A) Representative Pr2 DNA methylation patterns from each group. Each circle represents a CG site, closed circles are methylated. Each vertical line represents a single clone. (B) Quantification of DNA methylation percentages for each CG site of the Pr2 promoter. Two independent experiments (*n* = 2/group) were performed.
