# Supplementary material for: Early Dietary Exposures Epigenetically Program Mammary Cancer Susceptibility through Igf1-Mediated Expansion of the Mammary Stem Cell Compartment
Source: Cells. 2022 Aug 17;11(16):2558. doi: 10.3390/cells11162558 (PMC9406400; doi:10.3390/cells11162558)
Supplement: Supplementary file 1 [file cells-11-02558-s001.zip › cells-1807171 proof sup .pdf]

Supplemental Materials and Methods

Mammary Epithelial Cell (MEC) Isolation

Fourth and fifth mammary gland pairs were resected from mice and minced with sterile scalpels until glands were rendered to a paste. The paste was dissociated using collagenase/hyaluronidase (Stem Cell Technologies, cat #07919) in a shaking incubator at 37°C, 230 rpm for 3 hours according to the manufacturer's protocol. Red blood cells were lysed by washing the pellet in a 4:1 (v/v) mixture of 0.8% ammonium chloride (NH_4Cl) and cold Hanks' Balanced Salt Solution (HBSS, GE Healthcare Life Sciences, cat# SH30268.02) supplemented with 2% fetal bovine serum (FBS). Cells were then treated with 2 ml of 0.25% Trypsin/EDTA solution (Stem Cell Technologies, cat #07901) and 2 ml of 5mg/mL dispase (Gibco, cat#17105-041) supplemented with 10U of DNase I. The single-cell suspension was filtered through a 40μM mesh cell strainer.

Flow Cytometry and Cell Sorting

Cells were stained for 30 minutes at 4 °C with fluorophore-conjugated antibodies at the specified concentrations shown in Supplemental Table S2. After staining, cells were washed with DPBS three times and resuspended in 1 mL of HBSS solution supplemented with 2% FBS. Propidium iodide (PI) (5ug/ml, Sigma, cat#P4147) was loaded into the cell suspension immediately before analysis, separating live and dead cells. CompBeads (BD BioSciences, cat#552845) were used as single color and unstained controls. Stained cells were loaded onto a Fortessa X20 flow cytometer (BD Biosciences) and 100,000 events were recorded for each sample. Data were analyzed using FlowJo software V10 (RRID: SCR_008520). For cell sorting, a FACSAria II flow cytometer (BD Biosciences) was used. Sorted cell populations were reanalyzed and found to be 94%-98% pure, and cell viability was above 85%.

Mammosphere Culture and Limiting Dilution Assay

Freshly dissociated MECs from the fourth and fifth pairs of mammary glands were plated in EpiCult™-B Basal Medium supplemented with 5% FBS, 10% EpiCult™-B Proliferation Supplement (Stem Cell Technologies, cat#05610), Human Epidermal Growth Factor (EGF, 10ng/mL, Stem Cell Technologies, cat #78006), Recombinant Human Basic Fibroblast Growth Factor (FGF, 10ng/mL, Stem Cell Technologies, cat #78003) and Heparin (4μg/mL, Stem Cell Technologies, cat#07980) for 24 hours, followed by another 24-hour incubation in serum-free EpiCult™-B Basal Medium. MECs were then trypsinized and plated at 10,000 cells/well in pHEMA-coated 24-well plates (Santa Cruz Biotechnology, cat# sc-253284) with mammosphere growth medium: DMEM/Ham's F-12 (Caisson Labs, cat#DFP17) supplemented with 1X B27 supplement (Gibco, cat#17504044), EGF (20ng/mL) and FGF (20ng/mL). After seven days, mammospheres (P1) were collected, dissociated into single cells with 0.25% Trypsin/EDTA solution (Stem Cell Technologies, cat #07901), and plated again at 10,000 cells/well. After seven days, mammospheres (P2) with a diameter larger than 50 μM were counted.

To perform the limiting dilution assay (LDA), P2 mammospheres were dissociated into single cells and plated into 96-well ultra-low attachment plates (Corning, VWR cat #29443-034) at dilutions ranging from 1 to 512 cells per well, with 8 technical replicates (wells) per dilution. After seven days, wells were scored for the presence or absence of mammospheres. To assess the frequency of mammosphere-initiating cells, an extreme limiting dilution analysis was performed as described previously, and pairwise differences between the groups were compared with likelihood ratio tests using the asymptotic chi-squared test approximation to the log-ratio. Four independent experiments (n = 4) were performed.

Conditioned Medium Assay

Mammary stromal cells (Lin^{-}CD24^{low}CD29^{low}) from 5-week-old animals were sorted by FACS and plated using DMEM/Ham's F-12 medium (Caisson Labs, cat#DFP17). For the first 24 hours of culture, the medium was supplemented with 10% fetal bovine serum (FBS) to allow cell recovery. Cells were then trypsinized, counted and plated into fresh, serum-free DMEM/Ham's F-12 medium. Supernatants, i.e., conditioned media (CM), from the different diet groups were collected after 24 hours. For generating stem-enriched MECs, primary MECs from 5-week-old LT-C animals were harvested and cultured to P2 mammosphere as described above. P2 mammospheres were then disaggregated and plated at 10,000 cells/well in pHEMA-coated 24-well plates using the previously generated CM. Recombinant IGF1 (Life Technologies, cat# PHG0071) and picropodophyllin (PPP, Santa Cruz, cat# sc-204008) were added to the CM at a final concentration of 7.5 nM and 10 μM, respectively, and dose-response was determined in Supplemental Figure S2D-E. DMSO vehicle was added as treatment control. After seven days, mammosphere numbers with a size larger than 50 μm were counted. Data from four independent experiments (n = 4) are shown.

RNA Extraction and RT-qPCR

Fourth and fifth pairs of mammary glands were harvested and homogenized. Total RNA was extracted using the Quick-RNA™ Miniprep Plus Kit (Zymo Research, cat #R1057) following the manufacturer's protocol, and then reverse transcribed using M-MLV Reverse Transcriptase (Promega, cat #M1701) according to manufacturer's instructions. RT-qPCR was performed on an ABI Prism 7900HT sequence detection system (Applied Biosystems, Life Technologies Corporation, Carlsbad, CA). Messenger RNA levels in mammary tissues were assessed using the following TaqMan Assay IDs: IGF1: Mm00439560_m1, IGFBP5: Mm00516037_m1 and TATA-binding protein (TBP): Mm01277042_m1 (Thermo Fisher Scientific). In mammary stromal cells, for the *Igf1* promoters and methyltransferases, RT-qPCR reactions were performed using PowerUp SYBR Green Master Mix (Applied Biosystem, cat #4367659) and the primers used are listed in Supplemental Table S3. Target gene expression was normalized to TBP. All assays, including target and reference genes, were run as triplicates on the same plate. The $2^{-\Delta\Delta Ct}$ method was used to calculate relative gene expression levels.

Western Blot Analysis

Snap-frozen abdominal/inguinal mammary glands were extracted with boiling 2x Laemmli sample buffer. Protein concentrations were determined using the Pierce™ BCA protein assay kit (Thermo Fisher Scientific, Cat #23225) according to manufacturer's protocol. Protein samples (60 μg) were resolved on SDS-PAGE gels and transferred onto 0.2μM PVDF membranes (EMD Millipore, cat #ISEQ00010). After blocking with 3% milk/TBST, membranes were probed with the primary antibodies, followed by the appropriate HRP-conjugated secondary antibody shown in Supplemental Table S2. Bands were detected using the ECL Plus kit (Amersham, GE Healthcare, Piscataway, NJ). Images were captured with a FluorChem M imager and quantified with the AlphaView SA software (ProteinSimple, San Jose, CA, USA). Signals of target protein bands were normalized to GAPDH bands of the same sample and then normalized to the control group to calculate fold changes.

Immunohistochemistry Staining

Paraffin-embedded tissue blocks were cut into 4 μm sections and were deparaffinized in xylene and rehydrated in graded alcohols. Antigen retrieval was performed with citrate buffer in a pressure cooker (Decloaking Chamber, Biocare Medical, Pacheco, CA, USA). Immunohistochemistry was performed using an automated platform (intelliPATH, Biocare Medical). Antibodies and dilutions are shown in Supplemental Table S2. K5 antibody was detected using a Rabbit-on-Rodent HRP-Polymer reagent (Biocare Medical, cat #RMR622) and K8 antibody was detected using a Rat HRP-Polymer reagent (Biocare Medical, cat #BRR4016). Diaminobenzidine chromogen (ImmPACT DAB, Vector Laboratories,

cat #SK-4105) was used to visualize sites of antibody-antigen interaction. Slides were counterstained with hematoxylin, and coverslipped. Quantification of images was performed using the Fiji software or blinded, manual counting of at least six high power fields.
