# Supplementary material for: Early Dietary Exposures Epigenetically Program Mammary Cancer Susceptibility through Igf1-Mediated Expansion of the Mammary Stem Cell Compartment
Source: Cells. 2022 Aug 17;11(16):2558. doi: 10.3390/cells11162558 (PMC9406400; doi:10.3390/cells11162558)
Supplement: Supplementary file 1 [file cells-11-02558-s001.zip › revise Supplemental figures.pdf]

Zheng et al. Supplemental Figure S1

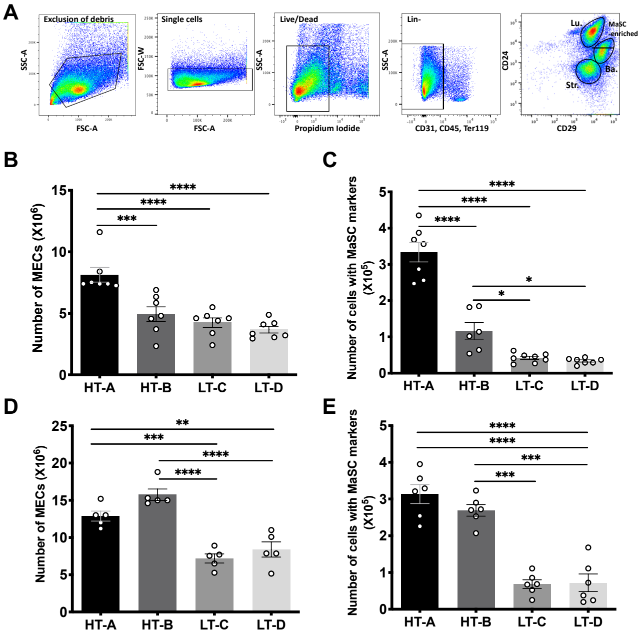

Supplemental Figure S1. The total number of MaSCs was increased in HT compared to LT animals. (A) Gating strategy for cell sorting. MaSC-enriched, basal (Ba.), luminal (Lu.) and stromal (Str.) cells were gated into Lin$^-$CD24^{med}CD29^{hi}, Lin$^-$CD24$^+$CD29^{hi}, Lin$^-$CD24^{hi}CD29^{low} and Lin$^-$CD24^{low}CD29^{low} subpopulations, respectively. **(B)** Total numbers of rudimentary MECs harvested from abdominal/inguinal mammary glands from 5-week-old animals (n=7/group). **(C)** Estimates of the total number of cells with MaSC markers in 5-week-old mice (n=7/group). The frequency of the MaSC-enriched population was multiplied by the number of MECs isolated from each corresponding sample. **(D)** Total numbers of rudimentary MECs harvested from abdominal/inguinal mammary glands from 10-week-old animals (n=5/HT-A; n=7/HT-B; n=4/LT-C; n=4/LT-D). **(E)** Estimates of the total number of cells with MaSC markers in 10-week-old mice (n=5/HT-A; n=7/HT-B; n=4/LT-C; n=4/LT-D). Mean ± SEM are shown. One-way ANOVA was used for statistical analysis, and pairwise comparisons were performed using Tukey's posttest, a≠b≠c, $P<0.05$.

Zheng et al. Supplemental Figure S2

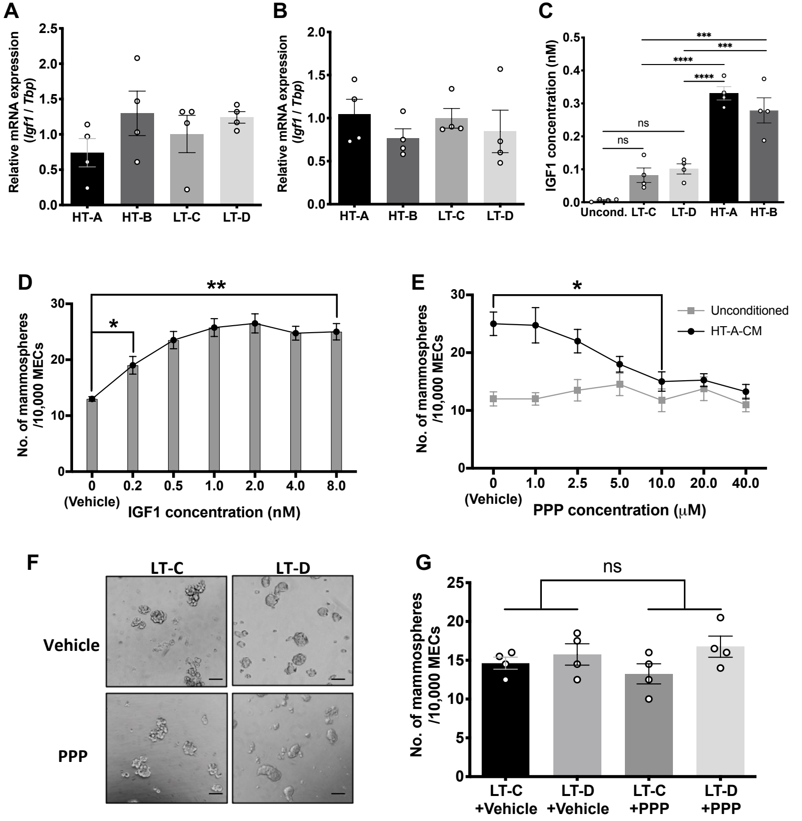

Supplemental Figure S2. (A) *Igf1* mRNA levels in FACS-sorted MaSC-enriched population and **(B)** luminal epithelial cells from mammary tissues of 5-week-old animals (n=4/group). Relative *Igf1* mRNA levels are expressed as fold change over group LT-C**. (C)** IGF1 protein concentrations in CM from HT and LT groups. Unconditioned DMEM/Ham's F-12 medium was used as a negative control. Four independent experiments (n=4) were performed. **(D)** Dose-response analysis to determine the effect of recombinant IGF1 on mammosphere numbers in LT-C-CM. **(E)** Dose-response analysis to determine the effect of picropodophyllin (PPP) on mammosphere numbers in unconditioned medium or in HT-A-CM. **(F)** Representative images and **(G)** bar graph showing numbers of mammospheres formed in LT-C-CM or LT-D-CM, supplemented with DMSO vehicle or 10 μM PPP. Scale bar =100 μm. Four independent experiments (n=4) were performed, and data are displayed as mean ± SEM. One-way ANOVA was used for statistical analysis and pairwise comparisons were performed using Tukey's posttest. a≠b, $P < 0.05$.

Zheng et al. Supplemental Figure S3

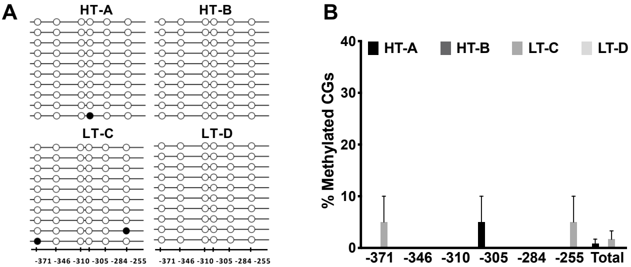

Supplemental Figure S3. *Igf1* Pr2 was not differentially methylated in mammary stromal cells from HT and LT groups. (A) Representative Pr2 DNA methylation patterns from each group. Each circle represents a CG site, closed circles are methylated. Each vertical line represents a single clone. **(B)** Quantification of DNA methylation percentages for each CG site of the Pr2 promoter. Two independent experiments (n=2/group) were performed.
